# An Improved Time-Frequency Analysis Method in Interference Detection for GNSS Receivers

**DOI:** 10.3390/s150409404

**Published:** 2015-04-21

**Authors:** Kewen Sun, Tian Jin, Dongkai Yang

**Affiliations:** 1 School of Computer and Information, Hefei University of Technology, Tunxi Road 193, Hefei 230009, China; 2 School of Electronic and Information Engineering, Beihang University, XueYuan Road 37, Beijing 100191, China; E-Mails: jintian@buaa.edu.cn (T.J.); edkyang@buaa.edu.cn (D.Y.)

**Keywords:** interference detection; time-frequency (TF) analysis; cross-terms; Wigner, Ville distribution (WVD); smoothed pseudo Wigner, Ville distribution (SPWVD); reassignment method

## Abstract

In this paper, an improved joint time-frequency (TF) analysis method based on a reassigned smoothed pseudo Wigner–Ville distribution (RSPWVD) has been proposed in interference detection for Global Navigation Satellite System (GNSS) receivers. In the RSPWVD, the two-dimensional low-pass filtering smoothing function is introduced to eliminate the cross-terms present in the quadratic TF distribution, and at the same time, the reassignment method is adopted to improve the TF concentration properties of the auto-terms of the signal components. This proposed interference detection method is evaluated by experiments on GPS L1 signals in the disturbing scenarios compared to the state-of-the-art interference detection approaches. The analysis results show that the proposed interference detection technique effectively overcomes the cross-terms problem and also preserves good TF localization properties, which has been proven to be effective and valid to enhance the interference detection performance of the GNSS receivers, particularly in the jamming environments.

## 1. Introduction

The demand for safety-critical applications (e.g., civil aviation, aircraft landing) using GNSSs has gained extensive and increased interest in recent years. With the advent of the new GNSSs, such as the European Union's Galileo system [1], the USA's modernized GPS [2] and China's Beidou/Compass [3], new radio navigation signals will be broadcast, and more attention has been devoted to the design of the signal structure, which is expected to increase the accuracy, availability, integrity and continuity of service, especially in the field of the safety of life (SOL) applications (e.g., the accuracy needed during the landing of an aircraft). High precision positioning and reliable SOL services represent the main challenges for the upcoming satellite navigation systems. The presence of disturbing signals, such as spurious, harmonic and electromagnetic interferences, will result in serious performance degradation for GNSS receivers.

Radio frequency interference (RFI) is one of the biggest threats for satellite navigation systems. Although the satellite navigation system has a certain capability to be immune from interference, since the direct sequence spread spectrum (DSSS) is utilized, due to the low power of the received GNSS signals, the presence of intentional or unintentional disturbing signals, such as spurious, harmonic and electromagnetic interferences, will result in serious performance degradation for GNSS receivers. Among all of the different error sources that can potentially corrupt GNSS signals, RFI is particularly harmful, since, in some cases, it cannot be mitigated by a simple correlation process [4]. The jamming environment is threatening for satellite navigation systems. Many systems rely on the transmission of radio frequency (RF) energy in the L-band. For example, the European Galileo E5a and E5b radio bands, located within 1164–1214 MHz, occupy frequencies already allocated for aeronautical radio navigation services (ARNS), such as tactical air navigation (TACAN), distance measuring equipment (DME) and secondary surveillance radar (SSR) [5]. In addition, other RF transmissions, such as continuous wave (CW) signals originating from the European Digital Video Broadcast Terrestrial (DVB-T) service, can be considered as the main threat for the GNSS signals, since they appear in Radio Navigation Satellite System (RNSS) frequency bands. The presence of RFI and other channel impairments can heavily degrade the reception of useful GNSS signals, which results in poor navigation accuracy or complete loss of tracking for GNSS receivers.

Currently, interference detection and mitigation (ID&M) have become very important issues for GNSS applications. Interference can be detected and mitigated through various means. On the hardware side, specialized instrumentation, such as choke rings or active beam-forming antennas, can be used to suppress interference and improve the reception of line of sight (LOS) satellite signals. The main drawbacks of these antenna techniques are their requirements for particular hardware configurations and their computational complexity [6–8]. In the GNSS receiver, a special front-end architecture design adopts pulse blanker or automatic gain control (AGC) to reduce interference. For example, a specific AGC and analog-to-digital converter (ADC) design performing digital pulse blanking has been implemented in the GPS L5 receiver [9]. In addition, a nonuniform ADC controlled by a digital AGC can be adaptively adjusted based on the interference power strength to obtain an optimized conversion gain of the ADC (*i.e.*, the SNR loss in ADC) and improved receiver performance [10,11]. However, these approaches require digital access to the feedback control of the AGC device, which is not a common output of generic analog front-ends used in the GNSS receivers.

In the literature, several interference mitigation techniques have been proposed and investigated, and each of them differs according to the operate domain (time, frequency or space). These techniques can be classified according to the specific processing domain. Considering time domain techniques, temporal filtering can be usually adopted. This method can be implemented at the digital intermediate frequency (IF) level after the ADC in the front-end of the GNSS receiver, which is effective only against narrow band RFI sources, because a wide band interference cannot be easily discriminated from the thermal noise by adopting temporal filtering. Frequency domain techniques are generally based on spectral estimation of the incoming signal, which is obtained by applying signal processing techniques, such as the discrete Fourier transform (DFT). These frequency domain techniques are typically performed by comparing the spectrum of the received signal with a theoretical threshold, which is usually determined according to a statistical model representing the received signal [12].

Recently, the research topic on transformed domain techniques (e.g., TF transform) has obtained increasing attention in the ID&M for GNSS receivers [13–16]. These techniques allow one to observe the received GNSS signals in a joint domain. In many cases, interferences may appear for a limited time and present a very variable behavior in frequency. In comparison to the GNSS signal, interferences are extremely different in terms of time and frequency characteristics. The presence of an interfering signal is limited to a region of the two-dimensional TF plane, and the adoption of the TF analysis is allowed to detect different types of disturbing signals. TF representations (TFRs) map a one-dimensional time signal into a two-dimensional function of time and frequency [17], which have found significant applications in non-stationary signal analysis. An interference mitigation technique based on the TFR approach has been described [18], where the TFR of the received GNSS signal is obtained by performing an orthogonal-like Gabor expansion on the samples at the output of the ADC of the GNSS receiver front-end. Another class of ID&M algorithm is reported aiming at obtaining a representation of the received signal in a different domain by making use of the time-scale transformation, which can be performed by means of wavelet transform [19].

Interfering signals are usually concentrated in a limited area of the TF plane, while noise is spread over the entire plane. The TF analysis approaches are very appealing as countermeasures in the detection of a large variety of interfering signals for GNSS receivers. There are different tools representing TF distributions, and the commonly-used TFRs in interference detection for GNSS applications include spectrogram, Wigner–Ville distribution (WVD) [13,16] and Choi–Williams transform (CWT) [16]. The spectrogram and WVD have been considered in the interference detection for GNSS applications [13]. The spectrogram approach presents the TF resolution trade-off problems according to the uncertainty principle, providing poor TF localization properties. In order to overcome the TF resolution trade-off problems of the spectrogram, WVD has been used in interference detection for GNSS receivers [13,16]. WVD is well known, since it provides nearly the best TF resolution among all of the TF distributions and also satisfies a large number of good properties [20], but it presents very severe cross-interfering terms without any physical meaning between true signal components (auto-terms), due to the interaction of different frequency components [16].

Most of the previous works on interference detection for GNSS receivers based on TF analysis are concerned with various TFR plots. Nevertheless, the quadratic TF distribution is usually a biased estimator for signal instantaneous frequency due to the presence of cross-term problems or makes a trade-off of temporal and frequency resolution due to the limitation of the uncertainty principle. In order to overcome or attenuate the cross-interfering terms present in the the quadratic TF distributions, the Choi–Williams distribution has been proposed to detect the sweep interference for GNSS receivers [16]. Appropriate image processing techniques can be also used for detection and parameter estimation of chirp signals by line detection in an image [21]. In addition, several kernel design methods have been proposed to mitigate the cross-term effect [22–24]. Unfortunately, these techniques need heavy computational complexity when they are applied in a real-time context. A reasonable method is to to introduce a window function in the time domain to reduce the undesired cross-term effects; therefore, the concept of the pseudo Wigner–Ville distribution (PWVD) is educed [20]. Consequently, the window function in PWVD can partially suppress the cross-terms to some ex0tent; the disadvantage of the filtering window operation is the degradation of the resolution, particularly in the frequency domain.

In PWVD, the time window operation is equal to frequency filtering in WVD, which can reduce the number of cross-interfering terms by suppressing the interferences between signal components sufficiently separated in time. In order to obtain a better readable result, the cross-terms between components in the frequency domain should be also minimized. Thus, an additional window function is added in order to perform a smoothing in time independently of the frequency smoothing. Therefore, the smoothed version of PWVD, namely the smoothed pseudo Wigner–Ville distribution (SPWVD), can be obtained [25]. The SPWVD is characterized by a separable kernel, which allows the time and frequency smoothing to be adjusted independently, which becomes one of the most versatile of Cohen's class TF distributions.

The smoothing windows can be adopted to reduce the cross-terms significantly; unfortunately, the SPWVD method also smears localized components, leading to less accurate localization of the signal auto-components in the TF plane compared to the WVD approach. Therefore, a reassignment method can be advantageously applied to improve TF localization properties in SPWVD [25,26]. In this way, the reassigned smoothed pseudo Wigner–Ville distribution (RSPWVD) can be obtained. The RSPWVD method is used to compensate for faults in mapping the TF energy distribution by relocating the value of the neighboring energy to the gravity center rather than the geometric center. In the RSPWVD method, by the adoption of the two-dimensional low-pass filtering smoothing function, the cross-term artifacts present in the quadratic TF distribution can be efficiently eliminated; meanwhile, by the use of the reassignment, the TF localization and aggregation properties of the auto-terms of the signal can be significantly improved.

In this paper, an improved TF analysis method by adopting RSPWVD has been proposed in interference detection for GNSS receivers. To the best of our knowledge, this interference detection technique based on joint TF analysis by adopting RSPWVD in the interference detection units for GNSS receivers is new. The performance of the proposed method has been deeply evaluated in comparison with the existing TF analysis approaches. Different localization properties and cross-term effects in the TF plane have been well investigated and compared among the aforementioned TF distributions adopted in interference detection for GNSS receivers.

In order to prove the effectiveness of the proposed TF analysis method by adopting RSPWVD in interference detection for GNSS receivers, an experiment is accomplished in the GPS L1 signal, which is characterized in additive white Gaussian noise (AWGN) corrupted by linearly-modulated sweep interference (chirp disturbance). The analysis results show that the proposed joint TF analysis by using RSPWVD eliminates the cross-terms significantly and preserves the high resolution of time and frequency in the TF plane at the same time. This developed improved TF analysis technique by adopting RSPWVD in interference detection makes the spectral characteristic of the interfering term sharply distinguishable among the received GNSS signal, which provides improved readability in the TF plane and enhanced detection performance for GNSS receivers with respect to the state-of-the-art TF analysis approaches.

## 2. Signal and System Model

The signal at the input of a GNSS receiver, in a noisy environment with RFI, can be written as:

(1) $$y_{RF}\left(t\right)=\sum_{i=1}^{N_{s}}r_{RF,i}\left(t\right)+\eta_{RF}\left(t\right)$$

*i.e.*, the sum of *N_s_* useful signals emitted by *N_s_* different satellites and of a disturbing term η*_RF_*(*t*) and *N_s_* is the number of satellites in view. The expression of the signal in space (SIS) transmitted by the *i*-th satellite and received at the GNSS receiver antenna with a propagation delay τ*_i_* is usually assumed as the following structure:

(2) $$r_{RF,i}\left(t\right)=A_{i}c_{i}\left(t-\tau_{i}\right)d_{i}\left(t-\tau_{i}\right)\cos\left[2\pi \left(f_{RF}+f_{d,i}\right)t+\varphi_{RF,i}\right]$$

where:

- *A_i_* is the amplitude of the *i*-th useful signal;
- τ*_i_* is the code phase delay introduced by the transmission channel;
- *c_i_*(*t* − τ*_i_*) is the pseudo random noise (PRN) code sequence, which is assumed to take a value in the set {−1,1};
- *d_i_*(*t* − τ*_i_*) is the bit stream of the navigation message, binary phase-shift keying (BPSK) modulated, including satellite data; and each binary unit is called a bit;
- *f_d,i_* is the Doppler frequency shift affecting the *i*-th useful signal, and φ*_RF,i_* is the initial carrier phase offset;
- *f_RF_* is the carrier frequency, and it depends on the GNSS signal band under analysis; in the case of the GPS L1 signal, *f_RF_* = *f_L_*_1_ = 1575.42 MHz.

In general, the disturbing term η*_RF_*(*t*) can be expressed as:

(3) $$\eta_{RF}\left(t\right)=j_{RF}\left(t\right)+w_{RF}\left(t\right)$$

where *j_RF_*(*t*) is a non-stationary RFI and *w_RF_*(*t*) is a zero-mean stationary AWGN process.

The interfering signal *j_RF_*(*t*) can assume different forms depending on the jammer that generates it. Several efforts have been devoted to the analysis and characterization of civilian GNSS jammers; despite significant differences, the transmitted jamming signal is usually frequency modulated with an almost constant amplitude. In this paper, the interference term *j_RF_*(*t*) is assumed to be in the class of sweep interference (linear chirp). Sweep interference is one of the main classes of the interfering signals, and its corresponding time-domain function represented by sinusoids can be written as follows:

(4) $$j_{RF}\left(t\right)=A_{\text{inst}}\left(t\right)\cos\left[2\pi f_{\text{inst}}\left(t\right)t+\varphi_{0}\right]$$

where *A_inst_*(*t*) is the interfering signal amplitude, *f_inst_*(*t*) denotes its instantaneous frequency and φ_0_ is the initial phase of the interference (at time *t* = 0), which can be assumed to be a random variable with a uniform distribution in the range [−*π*, +*π*).

In a linear chirp, the instantaneous frequency *f_inst_*(*t*) of the interfering signal evolves linearly with time over the interval [*f_RF_*+∆*f*_0_, *f_RF_*+∆*f*_1_], where *f_RF_* is the GNSS signal center frequency. Therefore, the instantaneous frequency *f_inst_*(*t*) can be expressed as:

(5) $$\begin{array}{cc} f_{\text{inst}}\left(t\right)=f_{0}+kt & 0\leq t\leq t_{j} \end{array}$$

where *f*_0_ is the starting frequency (at time *t* = 0), *f*_0_ = *f_RF_* + *∆f*_0_, *t_j_* is the frequency sweep period of the jamming signal and *k* is the rate of frequency increase or chirp rate, written as:

(6) $$k=\frac{f_{1}-f_{0}}{t_{j}}=\frac{\Delta f_{1}-\Delta f_{0}}{t_{j}}$$

where *f*_1_ is the final frequency during a specific frequency sweep period, *f*_1_ = *f_RF_* + ∆*f*_1_ and ∆*f*_1_ − ∆*f*_0_ stands for the frequency sweep.

The input signal *y_RF_*(*t*) defined in Equation (1) is received by the receiver antenna, down-converted and filtered by the receiver front-end. Then, the received signal before the analog to digital (A/D) conversion can be written as:

(7) $$\begin{array}{ll} y\left(t\right) & =\overset{N_{s}}{\underset{i=1}{\text{∑}}}r_{i}\left(t\right)+\eta \left(t\right)\\ & =\overset{N_{s}}{\underset{i=1}{\text{∑}}}A_{i}{\tilde{c}}_{i}\left(t-\tau_{i}\right)d_{i}\left(t-\tau_{i}\right)\cos\left[2\pi \left(f_{IF}+f_{d,i}\right)t+\varphi_{i}\right]+\eta \left(t\right) \end{array}$$

where *f_IF_* is the receiver intermediate frequency (IF). The term *c̃_i_*(*t* − τ*_i_*) represents the spreading sequence after filtering of the front-end, and here, the simplifying condition:

(8) $${\tilde{c}}_{i}\left(t\right)\approx c_{i}\left(t\right)$$

is assumed and the impact of the front-end filter is neglected. η(*t*) is the down-converted and filtered disturbing component, η[*t*] = η[*t*] + *w*[*t*].

Considering the interference term *j*[*t*], the mean power of the sweep interference can be defined as:

(9) J=Var{j[t]}

The jammer-to-noise ratio (JNR) is defined as follows:

(10) $$\frac{J}{N}=\frac{J}{\sigma_{IF}^{2}}=\frac{J}{N_{0}B_{IF}}$$

In order to avoid the cross-terms resulting from the interaction between the positive and negative frequency parts of the spectrum, the analytic representation of the received signal is adopted, provided as follows:

(11) $$y_{a}\left[t\right]=y\left[t\right]+jŷ\left[t\right]$$

where the analytic signal *y_a_*[*t*] has a real part *y*[*t*] and an imaginary part *y*[*t*], which contains the Hilbert transform of *y*[*t*]. The imaginary part is a version of the original real part with a 90° phase shift. The use of the analytic signal has two advantages: first, interference between positive and negative frequencies can be eliminated, as the analytic signal *y_a_*[*t*] has components belonging only to the half plane of positive frequencies; second, even though this filtering suppresses negative frequencies and the sampling rate is reduced, it does not introduce any loss of information.

## 3. Time Frequency Transforms

The classical method for analyzing a signal with time-varying frequency content is to split the time-domain signal into many segments. The signal to be transformed is multiplied by a window function, which is nonzero for only a short period of time, and then, take the Fourier transform of each segment as the window slid along the time axis, resulting in a two-dimensional representation of the signal. This is known as the short-time Fourier transform (STFT) operation, which is the most widely-used method for analyzing non-stationary signals. Additionally, simply, in the continuous time case, it is defined as:

(12) $$\text{STFT}\left(t,\omega \right)=\int_{-\infty }^{+\infty }y_{a}\left(\tau \right)h\left(\tau -t\right)e^{-j\omega \tau }d\tau$$

where *h*(*t*) is the analysis window, which is commonly a real and even window function centered on zero, *y_a_*(*t*) is the defined analytical signal to be transformed and *STFT*(*t*, ω) is a function of *t* and ω, which is linear and depends on the chosen window *h*.

In order to understand the time properties at a particular frequency, the definition of the STFT can also be expressed in the frequency domain by manipulating Equation (12), obtaining the following result:

(13) $$\text{STFT}\left(t,\omega \right)=\frac{1}{2\pi }e^{-j\omega t}\int_{-\infty }^{+\infty }Y_{a}\left(\omega^{\prime }\right)H\left(\omega^{\prime }-\omega \right)e^{j\omega^{\prime }t}d\omega^{\prime }$$

where *H*(ω) is the frequency window function, which is the Fourier transform of *h*(*t*). The dual relationship between Equation (12) and (13) is apparent; the TFR can be generated via a moving window in time or a moving window in frequency.

In the STFT analysis, one intends to achieve both high time and frequency resolution if possible. However, the resolution in the time domain is limited by the width of the window function *h*(*t*); similarly, the resolution in the frequency domain is limited by the width of the frequency window *H*(ω). As a result, the choice of a window to represent the signal by its spectrogram imposes a compromise between the conversation of temporal localization and that of frequency localization. This compromise is due to Heisenberg's uncertainty principle, which states that the window width in time and the window width in frequency are inversely proportional to each other. Therefore, choosing a small time window leads to good resolution in time and necessarily implies poor resolution in frequency; conversely, a long time window yields poor time resolution, but good frequency resolution. The length of the window plays a fundamental role in this TF compromise.

The squared magnitude of the STFT, denoted by *SPEC*(*t*, ω), is called spectrogram, which can be written as follows:

(14) $$\text{SPEC}\left(t,\omega \right)={\left|\text{STFT}\left(t,\omega \right)\right|}^{2}$$

where *STFT*(*t*, ω) is the STFT defined in Equation (12). The spectrogram of the signal has poor TF localization properties due to the presence of analysis window function.

In order to avoid the TF resolution trade-off problem of the spectrogram, WVD is adopted in interference detection for GNSS receivers. In the WVD, a time-dependent instantaneous auto-correlation function is chosen as:

(15) $$R\left(t,\tau \right)=y_{a}(t+\frac{\tau }{2})y_{a}^{*}(t-\frac{\tau }{2})$$

The WVD of *y_a_*(*t*) is then defined as the Fourier transform of this time-dependent instantaneous auto-correlation function [27], written as follows:

(16) $$\text{WVD}\left(t,\omega \right)=\frac{1}{2\pi }\int_{-\infty }^{+\infty }R\left(t,\tau \right)e^{-j\omega \tau }\text{d}\tau =\frac{1}{2\pi }\int_{-\infty }^{+\infty }y_{a}(t+\frac{\tau }{2})y_{a}^{*}(t-\frac{\tau }{2})e^{-j\omega \tau }\text{d}\tau$$

where *y_a_*(·) denotes the signal to be analyzed, *t* is the time, ω represents the angular frequency, τ is called the lag variable and (*) denotes the complex conjugate.

In addition, this class of bilinear (or quadratic) TF distributions can be most easily understood in terms of the ambiguity function. If the inverse Fourier transform of the instantaneous auto-correlation function *R*(*t*, τ) is taken with respect to *t* instead of τ, the ambiguity function can be obtained as follows:

(17) $$AF\left(\tau ,\theta \right)=\int_{-\infty }^{+\infty }R\left(t,\tau \right)e^{j\theta t}\text{d}t=\int_{-\infty }^{+\infty }y_{a}(t+\frac{\tau }{2})y_{a}^{*}(t-\frac{\tau }{2})e^{j\theta t}\text{d}t$$

The ambiguity function can be used to monitor the disturbing effect in the received GNSS signals.

The WVD has a number of desirable properties that make it a good indicator of how the energy of the signal can be viewed as a function of time and frequency. First, the WVD of any signal is always real. Second, it satisfies the time marginal condition:

(18) $$\int_{-\infty }^{+\infty }\text{WVD}\left(t,\omega \right)\text{d}\omega ={\left|y_{a}\left(t\right)\right|}^{2}$$

That is, by summing the TF distribution over all frequencies, the instantaneous energy of the signal at a particular time instant can be obtained. Similarly, the WVD also satisfies the frequency marginal condition:

(19) $$\int_{-\infty }^{+\infty }\text{WVD}\left(t,\omega \right)\text{d}t={\left|Y_{a}\left(\omega \right)\right|}^{2}$$

In this case, by summing the TF distribution over all time, the power spectrum of the signal at a particular frequency can be obtained.

Although WVD has many good properties and provides nearly the best resolution among all of the TF techniques, its main drawback comes from undesirable cross-term interference. The WVD is said to be bilinear, because the analyzed signal enters twice in its calculation. Consider the signal *y*(*t*) = *y*_1_(*t*) + *y*_2_(*t*), where *y*(*t*), *y*_1_(*t*) and *y*_2_(*t*) are analytic. Expanding the instantaneous auto-correlation function of *y*(*t*), we can obtain:

(20) $$R_{y}\left(t,\tau \right)=R_{y_{1}}\left(t,\tau \right)+R_{y_{2}}\left(t,\tau \right)+R_{y_{1}y_{2}}\left(t,\tau \right)+R_{y_{2}y_{1}}\left(t,\tau \right)$$

where *R_y_*__1__*_y_*__2__(*t*, τ) and *R_y_*__2__*_y_*__1__(*t*, τ) are the instantaneous cross-correlation functions (e.g., $R_{y_{1}y_{2}}\left(t,\tau \right)=y_{1}\left(t+\frac{\tau }{2}\right)y_{2}^{*}\left(t-\frac{\tau }{2}\right))$. Taking Fourier transforms of Equation (20) with respect to τ, it is easy to know that:

(21) WVDy(t,ω)=WVDy1(t,ω)+WVDy2(t,ω)+2Re{WVDy1y2(t,ω)}

where *WVD_y_*__1__(*t*, ω) and *WVD_y_*__2__(*t*,ω) are the WVDs of *y*_1_(*t*) and *y*_2_(*t*), respectively, and the last term is the cross-WVD (XWVD) between *y*_1_(*t*) and *y*_2_(*t*), provided as:

(22) $$WVD_{y_{1}y_{2}}\left(t,\omega \right)=\frac{1}{2\pi }\int_{-\infty }^{+\infty }y_{1}(t+\frac{\tau }{2})y_{2}^{*}(t-\frac{\tau }{2})e^{-j\omega \tau }\text{d}\tau$$

Thus, the WVD of the sum of two signals is not the sum of their corresponding WVDs, but also of their XWVDs. This means that the spectrum energy density of the sum of two signals does not reduce to the sum of the individual densities (unless the signals are spectrally disjoint). If *y*_1_(*t*) and *y*_2_(*t*) are mono-component signals, *WVD_y_*__1__(*t*,ω) and *WVD_y_*__2__(*t*,ω) are the auto-terms, while 2Re{*WVD_y_*__1__*_y_*__2__(*t*, ω)} is a cross-term.

**Figure 1:**
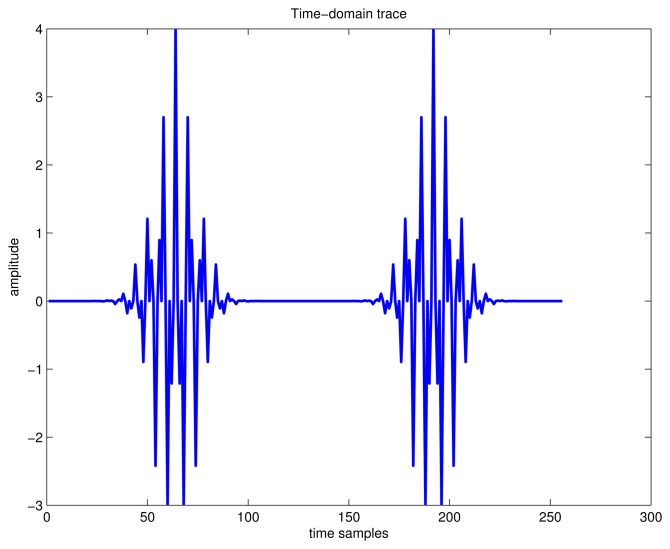
Time-domain trace of four Gaussian components' signal.

**Figure 2:**
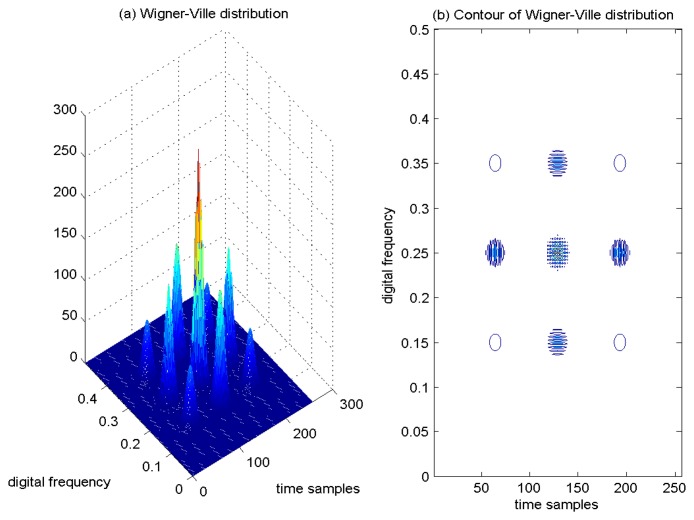
Wigner–Ville distribution of four Gaussian components' signal.

As a result, if a signal contains more than one component, in the TF plane, its WVD suffers from spurious features containing cross-terms that occur halfway between each pair of auto-terms. The magnitude of these oscillatory cross-terms can be twice as large as the auto-terms, and they do not possess any physical meaning. As an example, the time-domain signal, which contains four Gaussian components, is shown in Figure 1. The WVD of this signal is provided in Figure 2a, and correspondingly, the contour of the computed WVD is presented in Figure 2b. From Figure 2, there exist four peaks, which respectively denote the corresponding auto-terms of the four Gaussian components in the TF plane; in addition, the WVD also presents six cross-terms, which occur between each pair of Gaussian signal components, and among them, two cross-terms overlap in the diagonal intersection point of the rectangle connected by the four vertices (*i.e.*, four Gaussian components in the joint TF plane). These extra cross-terms have large oscillating amplitudes due to the interaction of the different signal components. The magnitudes of the oscillatory cross-terms can be twice as large as the auto-terms, but in the diagonal intersection position, the intensity of the corresponding cross-term can be four-times as large as the auto-terms, since two cross-terms overlap in this intersection point.

In Figure 2b, the serious cross-terms apparently present in the regions of the TF plane where we expect no energy at all, which make proper interpretation impossible. This is the main drawback of the WVD approach.

A feasible method to depress the effect of the cross-terms is to introduce windows in WVD; therefore, the concept of PWVD is educed. The WVD weighs equally all times of the future and past for a given time, but in the practical calculation of the distribution for a time instant *t*, we may concentrate the properties of the signal near the time of interest rather than from minus to plus infinity. Therefore, if we want to emphasize the signal around time *t*, a window function *h*(τ) can be multiplied with the instantaneous ambiguity function $y_{a}\left(t+\frac{\tau }{2}\right)y_{a}^{*}\left(t-\frac{\tau }{2}\right)$ in order to define the PWVD, which is the WVD windowed in the time direction [20], written as follows:

(23) $$\text{PWVD}\left(t,\omega \right)=\frac{1}{2\pi }\int_{-\infty }^{+\infty }h\left(\tau \right)y_{a}(t+\frac{\tau }{2})y_{a}^{*}(t-\frac{\tau }{2}){\text{e}}^{-j\omega \tau }\text{d}\tau$$

where *PWVD*(*t*,ω) is controlled by the window *h*(τ), which is an even function and peaked around *τ* = 0.

**Figure 3:**
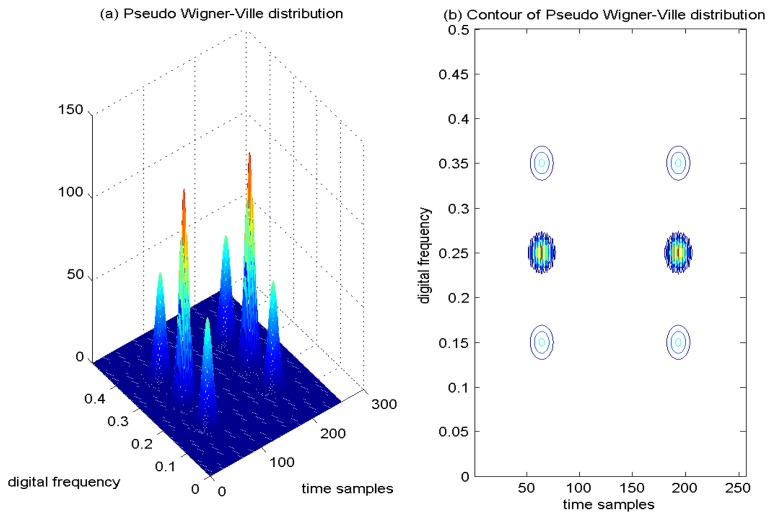
Pseudo Wigner–Ville distribution of four Gaussian components' signal.

Since the WVD is highly nonlocal, the window function is adopted in the PWVD to make it local. The overlapped window function contributes the cross-term suppression for multi-component signals. The operation of time-window overlay is equal to the frequency filtering in WVD; consequently, the interference between time-shifted signals is usually attenuated. An example of PWVD of the four Gaussian components signal is provided in Figure 3a, and correspondingly, the contour of the computed PWVD is presented in Figure 3b. From the results, it is easy to find that only two cross-terms remain in the TF plane, and the number of the extra cross-terms decreases from six to two, since the the cross-interfering term between each pair of time-shifted signal components is attenuated. In addition, from Figure 3b, it is clear to observe that the TF localization properties of the auto-terms of the analyzed signal are deteriorated in comparison with the WVD approach. In the PWVD, the cross-interfering terms have been partially mitigated by the adoption of the window function. The time window operation is equal to frequency filtering in WVD, which can reduce the number of cross-interfering terms by suppressing the interferences between signal components sufficiently separated in time. However, these advantages are achieved at the price of a blurring of the auto-terms of the signal and a loss of many desirable theoretical properties.

## 4. Interference Detection Based on Joint TF Analysis by Using RSPWVD

Although the WVD has many good properties and provides nearly the best resolution among all of the TF analysis techniques, due to its intrinsic quadratic nature, it suffers from cross-term interference when it is applied to multi-component signals. This drawback severely hinders the usefulness of the WVD for detecting RFI characteristics in the TF plane. In Section 3, the PWVD has been used to suppress the cross-terms for multi-component signals; however, many desirable properties of the WVD, such as marginals and instantaneous frequency, are annihilated, and the TF concentration property is also attenuated. In order to eliminate the cross-terms present in the quadratic TF distribution and to preserve good time and frequency resolution at the same time, a reassigned SPWVD method has been proposed in interference detection for GNSS receivers.

### 4.1. SPWVD

The unsatisfactory results obtained with the existing TF distributions justify the search for better tools; one way of achieving this is to start from the general form of quadratic representations. All of these existing TF distributions could be written in a generalized form, which can be used to facilitate the design of desirable TF transforms. This class of transform is known as Cohen's class [20], and the definition of Cohen's class of bilinear (or quadratic) TF distributions can be written as follows:

(24) $$\begin{array}{ll} C\left(t,\omega \right) & =\frac{1}{4\pi^{2}}\int_{-\infty }^{\infty }\int_{-\infty }^{\infty }AF\left(\tau ,\theta \right)g\left(\tau ,\theta \right){\text{e}}^{-j\left(\theta t+\omega \tau \right)}\text{d}\theta \text{d}\tau \\ & =\frac{1}{4\pi^{2}}\int_{-\infty }^{\infty }\int_{-\infty }^{\infty }\int_{-\infty }^{\infty }y_{a}(\text{s}+\frac{\tau }{2})y_{a}^{*}(\text{s}-\frac{\tau }{2})g\left(\tau ,\theta \right){\text{e}}^{-j\left[\theta \left(t-s\right)+\omega \tau \right]}\text{d}s\text{d}\theta \text{d}\tau \end{array}$$

where *AF*(τ, θ) is the ambiguity function defined in Equation (17); and *g*(τ, θ) is a two-dimensional parametrization function defined in the ambiguity function domain, which is called the kernel function of Cohen's class. This kernel function determines the properties of the bilinear TF distribution, which is often a low-passing function and normally serves to mask out the interference in the original Wigner–Ville representation. When considering *g*(τ, θ) = 1, WVD can be obtained.

Cohen's class can be also rewritten as the double convolution of the WVD of the signal *y_a_*(*t*) and a two-dimensional smoothing function, provided as follows:

(25) $$\begin{array}{ll} C\left(t,\omega ;\Pi \right) & =W_{ya}\left(t,\omega \right)**\Pi \left(t,\omega \right)\\ & =\frac{1}{4\pi^{2}}\int_{-\infty }^{\infty }\int_{-\infty }^{\infty }\prod \left(t-s,\omega -\theta \right)W_{y_{a}}\left(s,\theta \right)\text{d}s\text{d}\theta \end{array}$$

(26) $$\prod \left(t,\omega \right)=\int_{-\infty }^{\infty }\int_{-\infty }^{\infty }g\left(\tau ,\theta \right)e^{-j\left(\omega \tau +\theta t\right)}\text{d}\tau \text{d}\theta$$

where (**) denotes the double convolution operation, Π(*t*, ω) is a two-dimensional smoothing function and *W_ya_*(*s*,θ) is the WVD of the signal *y_a_*(*t*). Different TFRs can be obtained from the fundamental WVD by applying a different smoothing function Π(*t*, ω). When considering WVD, Π(*t*, ω) = δ(*t*)δ(ω).

If a separable smoothing function is considered, it can be written as the product of windows from both time and frequency domains:

(27) ∏(t,ω)=g(t)H(−ω)

where *H*(ω) is the Fourier transform of the window function *h*(*t*), which allows the smoothing of the cross-interfering terms oscillating in parallel with the frequency axis (frequency smoothing); and the window function *g*(*t*) allows the smoothing of the cross-interfering terms oscillating in parallel with the temporal axis (temporal smoothing). Obviously, the wider the spreading, the more the smoothing. Therefore, the smoothing in the ambiguity function domain combined with the parametrization function allows both the suppression of the cross-terms and the preservation of the auto-ambiguity terms of the analyzed signal.

Since the cross-terms with the WVD are strongly oscillating, the most effective way of removing cross-term interference is to apply two-dimensional low-pass filtering in the ambiguity domain. The resulting two-dimensional convolution of the WVD in Equation (25) defines the smoothed version of PWVD, that is SPWVD, which can be written as follows:

(28) $$\text{SPWVD}\left(t,\omega ;g,h\right)=\int_{-\infty }^{\infty }h\left(\tau \right)\int_{-\infty }^{\infty }g\left(t^{\prime }-t\right)y_{a}(t^{\prime }+\frac{\tau }{2})y_{a}^{*}(t^{\prime }-\frac{\tau }{2})e^{-j\omega \tau }\text{d}t^{\prime }\text{d}\tau$$

Therefore, an independent and progressive control can be applied to the WVD in both time and frequency directions. The independency of *h*(*t*) and *g*(*t*) makes SPWVD more flexible to reduce the cross-terms present in WVD.

### 4.2. Reassignment Method

Compared with WVD, SPWVD can be used to effectively depress the influence of the cross-interfering terms of a multi-component signal, but its TF concentration and localization properties decrease somewhat. In order to improve the TF aggregation properties in SPWVD, a reassignment method has been considered.

From Equation (25), we can know that the two-dimensional smoothing function Π(*t* − *s*, ω − θ) determines a certain TF region at the neighborhood nearby the point (*t*, ω), inside which a weighted average of the WVD *W_ya_*(*s*, θ) of the signal *y_a_*(*t*) is performed. However, these mean values may not be symmetrically distributed around a certain point (*t*, ω), which is the geometrical center of the TF region. Consequently, the point (*t*, ω) is not truly representative for such a region. In contrast, the energy gravity center of such a region is more approximate to represent the local energy distribution of the analyzed signal. The local energy distribution Π(*t* − *s*, ω − θ)*C*(*s*, θ; Π) of Cohen's class TF distribution can be assumed as the distribution of mass, and it is better to assign the total mass to the gravity center rather than to its geometrical center.

In this paper, the reassignment method is considered to relocate each value of Cohen's class distribution *C*(*t*, ω) at any point (*t*, ω) to another point (*t̂*, ω̂), which is the gravity center of the signal's energy distribution around the point (*t*, ω). Then, the reassigned Cohen's class TF distribution can be defined as follows:

(29) $$C^{\left(r\right)}\left(t^{\prime },\omega^{\prime };\prod \right)=\int_{-\infty }^{\infty }\int_{-\infty }^{\infty }C\left(t,\omega ;\prod \right)\delta \left(t^{\prime }-\hat{t}\left(y_{a};t,\omega \right)\right)\delta \left(\omega^{\prime }-\hat{\omega }\left(y_{a};t,\omega \right)\right)\text{d}t\text{d}\omega$$

where:

(30) $$\hat{t}\left(y_{a};t,\omega \right)=\frac{\int_{-\infty }^{\infty }\int_{-\infty }^{\infty }s\prod \left(t-s,\omega -\theta \right)W_{y_{a}}\left(s,\theta \right)\text{d}s\text{d}\theta }{\int_{-\infty }^{\infty }\int_{-\infty }^{\infty }\prod \left(t-s,\omega -\theta \right)W_{y_{a}}\left(s,\theta \right)\text{d}s\text{d}\theta }$$

(31) $$\hat{\omega }\left(y_{a};t,\omega \right)=\frac{\int_{-\infty }^{\infty }\int_{-\infty }^{\infty }\theta \prod \left(t-s,\omega -\theta \right)W_{y_{a}}\left(s,\theta \right)\text{d}s\text{d}\theta }{\int_{-\infty }^{\infty }\int_{-\infty }^{\infty }\prod \left(t-s,\omega -\theta \right)W_{y_{a}}\left(s,\theta \right)\text{d}s\text{d}\theta }$$

*C*^(^*^r^*^)^(*t′*, ω′; Π) is Cohen's class TF distribution after reassignment. Therefore, if a suitable smoothing kernel is selected, all of the bilinear TF distributions after reassignment are able to mitigate the cross-terms and keep high TF concentration properties at the same time. In particular, when the reassignment method is applied to SPWVD, RSPWVD can be obtained, which is calculated as follows:

(32) $$\text{RSPWVD}\left(t^{\prime },\omega^{\prime };g,h\right)=\int_{-\infty }^{\infty }\int_{-\infty }^{\infty }\text{SPWVD}\left(t,\omega ;g,h\right)\delta \left(t^{\prime }-\hat{t}\left(y_{a};t,\omega \right)\right)\cdot \delta \left(\omega^{\prime }-\hat{\omega }\left(y_{a};t,\omega \right)\right)\text{d}t\text{d}\omega$$

where:

(33) $$\hat{t}\left(y_{a};t,\omega \right)=t-\frac{\text{SPWVD}\left(t,\omega ;\tau_{g},h\right)}{2\pi \text{SPWVD}\left(t,\omega ;g,h\right)}$$

(34) $$\hat{\omega }\left(y_{a};t,\omega \right)=\omega +j\frac{\text{SPWVD}\left(t,\omega ;g,D_{h}\right)}{2\pi \text{SPWVD}\left(t,\omega ;g,h\right)}$$

with τ*_g_* = *g*(*t*) and $D_{h}(t)=\frac{\text{d}h(t)}{\text{d}t}$.

**Figure 4:**
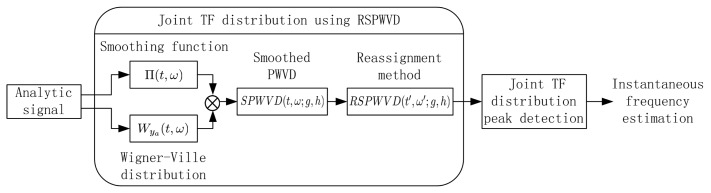
Joint TF distribution based on reassigned smoothed pseudo Wigner–Ville distribution (RSPWVD) in interference detection for GNSS receivers.

The RSPWVD *RSPWVD*(*t′*, ω′; *g*, *h*) can be used to eliminate the cross-term artifacts inherent in the quadratic TF distributions, which presents good resolution in both the time and frequency domains; therefore, in this work, the joint TF analysis based on RSPWVD has been firstly proposed in interference detection for GNSS receivers, which is clearly illustrated in Figure 4. The double convolution of the WVD *W_y_a__*(*t*, ω) of the analytical signal and the two-dimensional smoothing function Π(*t*, ω) is performed to obtain the *SPWVD*(*t*, ω; *g*, *h*); after reassignment, the cross-term-free TF distribution *RSPWVD*(*t′*, ω′; *g*, *h*) has good readability, which is suitable for multi-component signal analysis. The proposed joint TF analysis by using RSPWVD makes the spectral characteristic of the interfering signal sharply distinguishable among the received GNSS signal; therefore, the instantaneous frequency of the disturbing term in the received GNSS signals can be effectively estimated by detecting the peaks of the proposed joint TF distribution.

## 5. Performance Evaluation

In this section, the performance of the proposed interference detection method based on joint TF analysis by using RSPWVD is analyzed. In particular, this proposed new algorithm, which adopts RSPWVD to detect sweep interference in GNSS receivers, is compared with the conventional interference detection approaches in the disturbing scenario.

**Figure 5:**
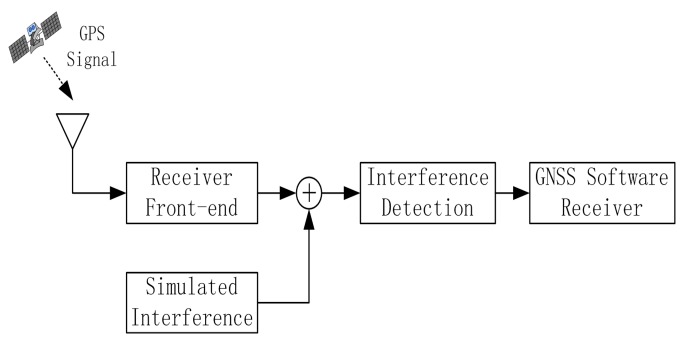
Scheme of the measurement test in the lab.

**Figure 6:**
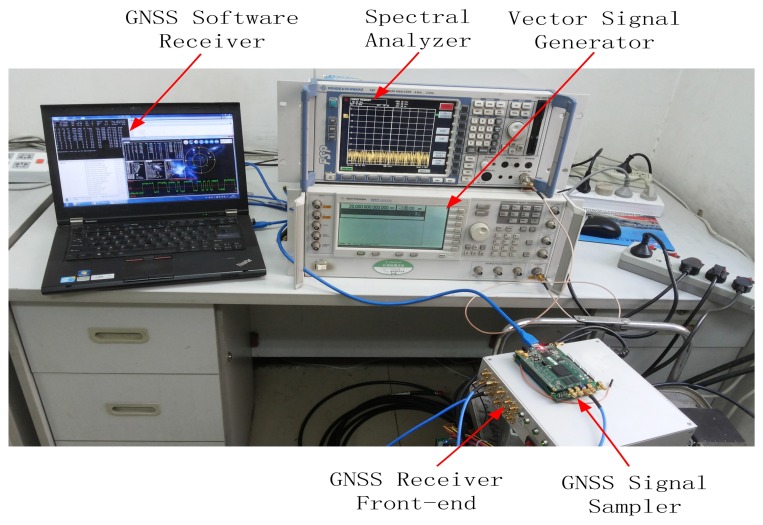
Experimental setup of the measurement test in the lab.

The mentioned interference detection approaches are tested on real GPS data collected by using the GNSS software receiver developed at Beihang University [28]. The scheme of the test is reported in Figure 5, while an image of the experimental setup adopted for collecting the GPS data corrupted by sweep interference is depicted in Figure 6. The real GPS samples are collected by using the GNSS software receiver connected to the Trimble Zephyr Geodetic 2 antenna placed on the roof of the new main building at Beihang University in an open-sky static condition; the developed software interferer is adopted for generating the sweep interfering signal for GNSS applications. The generated sweep interference is added to the GPS samples collected by the GNSS receiver front-end.

In the experiment, the scenario adopted for the test is characterized by the setting parameters provided in Table 1, representing the real GPS L1 signal in zero mean Gaussian noise corrupted by a constant amplitude linearly frequency modulated interference (linear chirp), which is commonly considered as a test bench in interference detection for GNSS receivers.

In the experiment settings, the JNR value of the sweep interference is set to be −1 dB; in a linear chirp, the instantaneous frequency *f_inst_* of the interfering signal evolves linearly with time over the interval [*f*_L1_ + ∆*f*_0_, *f*_L1_ + ∆*f*_1_], where *f*_L1_ is the GPS L1 signal center frequency, ∆*f*_0_ = +7 MHz and ∆*f*_1_ = −9 MHz.

**Figure 7:**
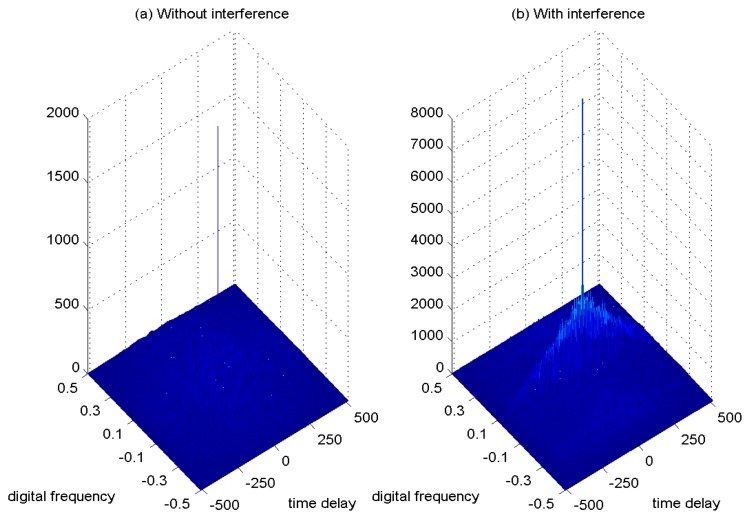
Ambiguity function of the GPS L1 signal. *C*/*N*_0_ = 46 dB-Hz. (**a**) Without interference; (**b**) with interference, jammer-to-noise ratio (JNR) = − 1 dB.

In Figure 7a, the ambiguity function of the GPS L1 signal without interference is shown as a spike. In the case with the presence of sweep interference, the ambiguity function of the interfered GPS L1 signal is depicted in Figure 7b, where the disturbing effect can be clearly observed. The adoption of the ambiguity function of the received interfering signal is beneficial to better monitor the interference contribution in the received GNSS signals.

**Figure 8:**
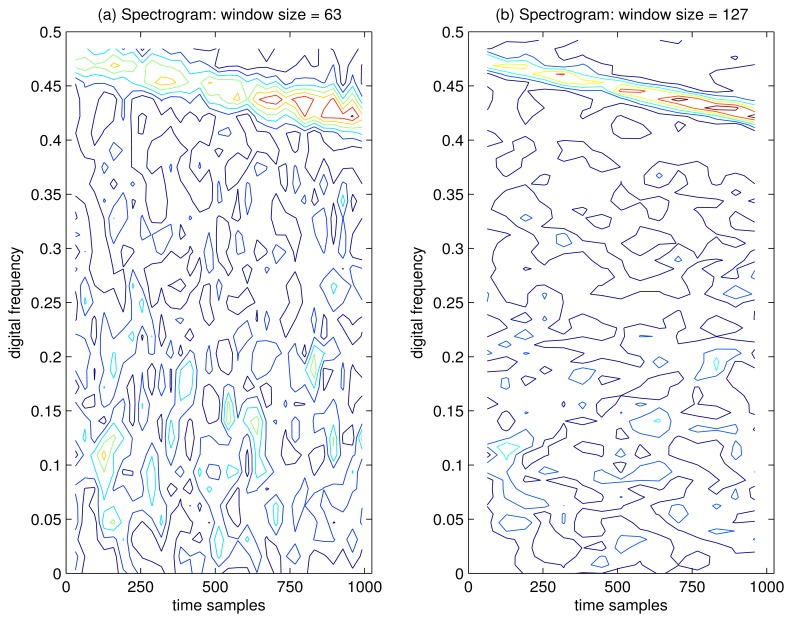
Spectrogram of the GPS L1 signal with sweep interference. The spectrogram has been evaluated by using a Hamming window. *C*/*N*_0_ = 46 dB-Hz, JNR = −1 dB.

In Figure 8, the spectrogram of the GPS L1 signal with sweep interference is depicted, where the Hamming window function is chosen. In Figure 8a, the window size is 63 samples, where the disturbing term more or less emerges in the TF plane, but very poor TF localization properties are obtained with this approach. In order to evaluate the TF characteristics of the spectrogram by increasing the length of the analysis window, the spectrogram of the interfered GPS L1 signal is presented in Figure 8b, where the window length is increased to 127 samples. It is clear that a long time window leads to improved frequency resolution and inevitably yields poor resolution in time. The spectrogram is nonlinear, but this nonlinearity results from the operation of squared magnitude and, therefore, does not lead to undesirable cross-terms present in the WVD. In practice, the spectrogram approach cannot be used to provide the instantaneous frequency estimate for the interfering signal, although sometimes, a good approximation can be achieved.

**Figure 9:**
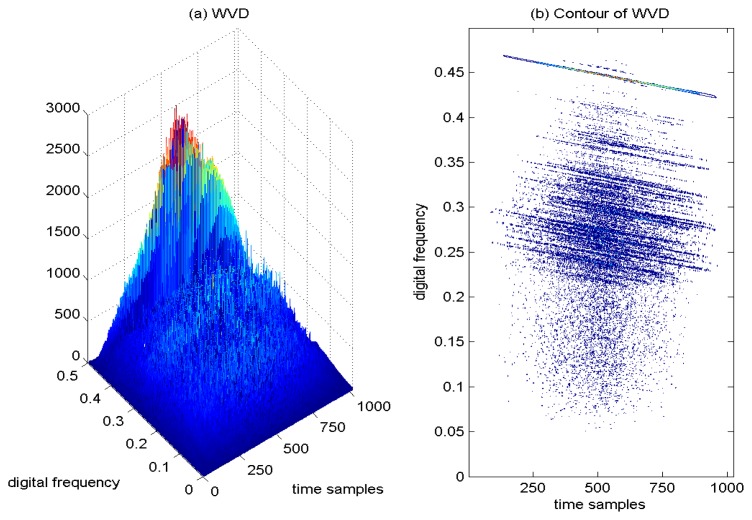
Wigner–Ville distribution of the GPS L1 signal with sweep interference. *C*/*N*_0_ = 46 dB-Hz, JNR = −1 dB.

In Figure 9a, the WVD of the GPS L1 signal with the sweep interference is depicted, and correspondingly, the contour of the computed WVD is presented in Figure 9b. The interfering term presents a linear behavior in frequency, which is well localized in the restricted portion of the TF plane. The price that is paid for high TF resolution with the WVD approach is the undesirable cross-terms. Due to the interaction of the different signal components, the presence of the serious cross-terms is apparently observed in Figure 9b, which makes interpretation quite difficult and brings much error for the estimation of the instantaneous frequency of the interfering signal.

**Figure 10:**
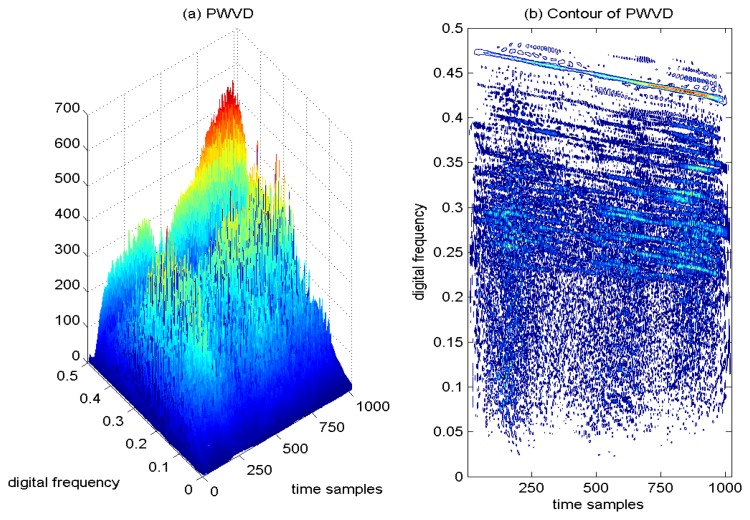
Pseudo Wigner–Ville distribution of the GPS L1 signal with sweep interference. *C*/*N*_0_ = 46 dB-Hz, JNR = −1 dB.

In Figure 10a, the PWVD of the GPS L1 signal with the sweep interference is depicted, and correspondingly, the contour of the computed PWVD is presented in Figure 10b. It is clear that although the sweep interfering term presents a linear behavior in the TF plane, the PWVD presents less accurate TF localization precision, since the TF concentration property of the PWVD is attenuated. In addition, the cross-terms can be still observed in the TF plane by using the PWVD approach.

**Figure 11:**
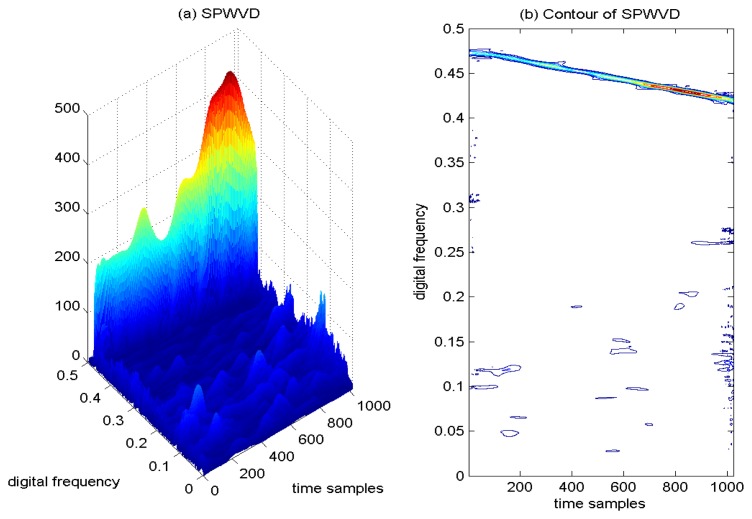
Smoothed pseudo Wigner-Ville distribution of the GPS L1 signal with sweep interference. *C*/*N*_0_ = 46 dB-Hz, JNR = −1 dB.

In order to mitigate the cross-interfering terms present in the WVD, a two-dimensional smoothing function is used to smooth out the interfering signal in both the time and frequency domains. In Figure 11a, the SPWVD of the interfered GPS L1 signal is provided, where the peaks of the SPWVD are clearly observed, denoting the sweep interference contribution. The corresponding contour of the SPWVD is provided in Figure 11b, and it is easy to find that the undesired sweep interference shows a linear behavior in frequency, which is localized in the linear portion of the TF plane. From Figure 11b, it is very clear that the undesired cross-interfering terms are partially mitigated by the adoption of the two-dimensional smoothing function in the SPWVD. Although the adoption of the smoothing window in SPWVD is beneficial to suppress the cross-terms in the TF plane, it smears localized components, leading to less accurate TF localization of the auto-terms of the signal, as compared to the WVD approach. The disadvantage of this filtering window operation is that it limits the original excellent TF resolution features.

**Figure 12:**
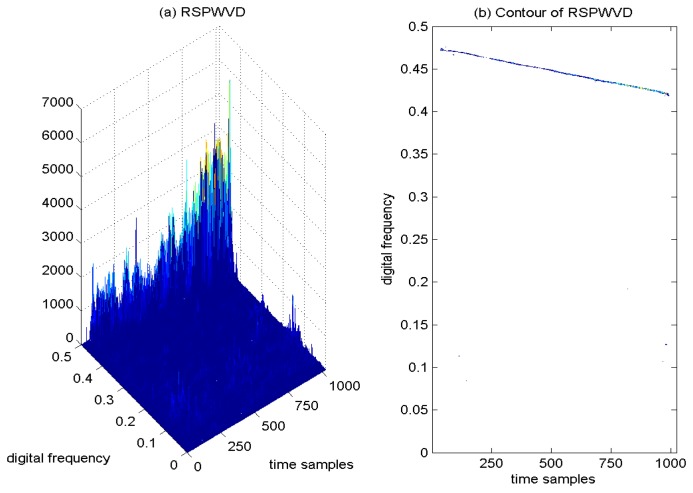
Joint TF analysis by adopting RSPWVD for the GPS L1 signal with sweep interference. *C*/*N*_0_ = 46 dB-Hz, JNR = −1 dB.

Therefore, in order to remove the cross-terms present in the quadratic TF distribution and to preserve the good TF energy concentration properties at the same time, an improved TF distribution by adopting RSPWVD has been proposed in interference detection for GNSS receivers. This proposed joint TF distribution of the interfered GPS L1 signal is depicted in Figure 12a, and the corresponding contour of the calculated TF distribution of RSPWVD is provided in Figure 12b. From the results, it is clear that the undesired sweep interfering term presents a very strict linear behavior in the TF plane with the proposed joint TF distribution based on RSPWVD, which provides representations that are easy to interpret in interference detection for GNSS receivers. This proposed improved TF analysis technique effectively eliminates the cross-term artifacts present in the quadratic TF distributions, which shows good readability in the TF plane. In the reassigned method, the smoothing window is moved from the geometrical center to the energy gravity center of the TF distribution. Therefore, with the proposed RSPWVD method, the disturbing interference is highly localized in the restricted region of the TF plane, and a much improved TF localization property can be achieved in comparison to the SPWVD approach. The proposed joint TF distribution based on RSPWVD has been proven to be valid and effective in interference detection for GNSS receivers in jamming environments.

This proposed joint TF analysis technique based on RSPWVD effectively solves the cross-term problem of the bilinear TF distribution and keeps good time and frequency resolution in the TF plane at the same time, which has been proven to be effective to adopt in interference detection for GNSS receivers. With this developed interference detection technique, the interfering signal can be correctly identified, and its instantaneous frequency can be precisely estimated. In the following interference excision/mitigation unit (anti-jamming device), the estimated instantaneous frequency of the interfering signal will be further used to control the coefficients of an excision filter (notch filter) that adaptively removes the disturbing interference signal.

## 6. Conclusions

In this paper, an improved TF analysis method based on RSPWVD has been proposed to detect sweep interference for GNSS receivers. In order to prove the advantages and effectiveness of the developed technique, a comprehensive performance comparison has been carried out compared with the existed TF analysis approaches. The experiments have been performed on GPS L1 signals in the disturbing scenario in order to support the theoretical analysis among the aforementioned TF distributions adopted in interference detection for GNSS receivers.

From the analysis results, the spectrogram approach presents the TF resolution trade-off problems and provides poor localization properties in the TF plane; the interference detection based on WVD presents a severe cross-interfering term problem due to the interaction of different frequency components, which makes proper disturbance interpretation impossible. Then, the SPWVD method is adopted to partially mitigate the cross-terms present in the bilinear TF distribution, since a two-dimensional smoothing function is added in both the time and frequency domains, but the disadvantage of using filtering windows is the degradation of time and frequency resolution.

In order to eliminate the cross-term problem and preserve good time and frequency resolution in the TF plane at the same time, an improved joint TF distribution by using RSPWVD has been firstly proposed in interference detection for GNSS receivers. The interference detection based on joint TF distribution by using RSPWVD efficiently combines the removal of the cross-interfering terms provided by a two-dimensional smoothing kernel function and an increased TF concentration of the auto-terms of the signal components achieved by the reassignment. From the analysis results, the proposed interference detection method based on RSPWVD successfully overcomes the cross-term problem and, meanwhile, presents good TF localization properties, which provide much improved interference detection performance in comparison with the existing TF analysis approaches.

The developed interference detection technique based on joint TF analysis by adopting RSPWVD has been proven to be suitable to improve the interference detection performance in the jamming environments, which is promising for adoption in the anti-interference GNSS receiver design for civil aviation and military purposes, particularly in disturbing environments.

## Figures and Tables

**Table 1:** Experimental setting parameters.

**Parameter**	**Value**
Carrier-to-noise ratio, *C*/*N*_0_	46 dB-Hz
Sampling frequency, *f_s_*	24 MHz
Intermediate frequency, *f_IF_*	40.42 MHz
Code length	1023 chips
Sweep period	1 ms
Spectrogram analysis window	Hamming
